# Retraction: Long noncoding RNA OIP5-AS1 targets Wnt-7b to affect glioma progression via modulation of miR-410

**DOI:** 10.1042/BSR20180395_RET

**Published:** 2026-08-07

**Authors:** Wei-Li Sun, Tian Kang, Yuan-Yu Wang, Jian-Ping Sun, Chen Li, Hong-Jiang Liu, Yue Yang, Bao-Hua Jiao

**Affiliations:** 1 Department of Rehabilitation, The Second Hospital of Hebei Medical University, Shijiazhuang, Hebei 050000, P.R. China; 2 Department of Pediatrics, the First Hospital of Shijiazhuang, Shijiazhuang 050011, China; 3 Department of Neurosurgery, The Second Hospital of Hebei Medical University, Shijiazhuang, Hebei 050000, P.R. China

**Keywords:** glioma, Invasion, Long noncoding RNA, microRNA, Migration, Proliferation

This article is being retracted from *Bioscience Reports* at the request of the Editor-in-Chief and the Editorial Board. This follows the receipt of a notification from a reader, alerting the Editorial Board to the unusual appearance of the western blots of Figure 3A, and to the high similarity between the Figure 3A GAPDH bands of this article and the Figure 4C GAPDH bands of Chen et al. 2019 (DOI: 10.22038/ijbms.2019.34853.8270). The authors have been contacted with regard to the retraction and have not responded to the Journal's queries or to the concerns. Given the extent of the issues raised, the Editorial Board stands by the decision to retract the article.

